# D‐Chiro‐Inositol and LPS Induce a PCOS‐Like Hyperandrogenic Response in Human KGN Granulosa Cells

**DOI:** 10.1111/jcmm.70779

**Published:** 2025-09-11

**Authors:** Cristiano Giuliani, Giovanni Casoli, Giovanna Di Emidio, Carla Tatone, Arturo Bevilacqua

**Affiliations:** ^1^ Department of Dynamic, Clinical Psychology and Health Studies Sapienza University of Rome Rome Italy; ^2^ Department of Life, Health and Environmental Sciences University of L'Aquila L'Aquila Italy; ^3^ The Experts Group on Inositol in Basic and Clinical Research, and on PCOS (EGOI‐PCOS) Rome Italy; ^4^ Systems Biology Group Lab and Research Center in Neurobiology Daniel Bovet (CRiN) Rome Italy

**Keywords:** aromatase, bacterial lipopolysaccharide, CYP19A1, D‐chiro‐inositol, hyperandrogenism, myo‐inositol, ovarian dysfunction, PCOS

## Abstract

Hyperandrogenism is a key hallmark of polycystic ovary syndrome, a prevalent endocrine disorder affecting women of reproductive age and often leading to infertility. We previously observed that high doses of D‐chiro‐inositol in mice reduce ovarian aromatase expression, contributing to a hyperandrogenic state. Given that similar effects have been reported in tumour‐derived human KGN granulosa cells treated with bacterial lipopolysaccharide, we investigated whether D‐chiro‐inositol could elicit a comparable hyperandrogenic response in these cells, thereby shedding light on aberrant mechanisms potentially involved in polycystic ovary syndrome. Using lipopolysaccharide and myo‐inositol as controls, we assessed KGN cells for proliferation, viability, inflammatory response, cellular and mitochondrial reactive oxygen species, expression of antioxidant enzyme genes, aromatase expression, and estradiol secretion. None of the treatments affected cell proliferation or viability. Both D‐chiro‐inositol and myo‐inositol showed anti‐inflammatory and antioxidant effects, whereas lipopolysaccharide induced inflammation and acted as a pro‐oxidant. Notably, D‐chiro‐inositol and lipopolysaccharide downregulated aromatase gene and protein expression, resulting in reduced estradiol secretion. In contrast, myo‐inositol had no significant impact on aromatase expression or oestrogen production. These findings suggest that D‐chiro‐inositol and lipopolysaccharide may serve as useful tools for probing the dysregulated molecular and cellular pathways associated with polycystic ovary syndrome, particularly those contributing to hyperandrogenism.

## 1 Introduction

Polycystic Ovary Syndrome (PCOS) is an endocrine disorder affecting young women, with an estimated prevalence of 10%–13% [1]; frequently associated with infertility and a reduced quality of life [2]. It has a multifactorial aetiology involving genetic, environmental, and familial components and is linked to insulin resistance, hormonal imbalances, oxidative stress, and chronic inflammation [3].

Among PCOS diagnostic signs [4], hyperandrogenism appears as one of the major defining characteristics [5] being present in most, although not all, patients [6]. Elevated androgen levels in PCOS are associated with inflammatory responses, including the expression of inflammatory cytokines within the ovaries, which can result in mitochondrial damage and contribute to follicular cell apoptosis, follicular dysplasia, and ovulatory dysfunction [7, 8]. A primary culprit of hyperandrogenism in PCOS is insulin resistance, which leads to hyperinsulinemia and, in turn, inhibition of synthesis of sex hormone‐binding globulin (SHBG) in the liver, and stimulation of androgen production in ovarian follicle theca cells [9, 10]. Increased androgen secretion by theca cells exacerbates PCOS symptoms and harms follicular development [11].

Granulosa cells (GCs) play an essential role in ovarian oestrogen production, folliculogenesis, and oocyte development [12], via the activity of the biosynthetic enzyme aromatase [13]. Aromatase dysregulation and subsequent abnormal oestrogen production lead to other hormonal imbalances contributing to PCOS and other diseases such as ovarian cancer [13].

Preclinical research into the aetiology and cellular mechanisms of PCOS increasingly employs mammalian in vivo animal models and in vitro cellular systems, among others [14, 15].

Our group has previously investigated the role of inositols in the pathophysiology of PCOS in in vivo murine models [16, 17]. Myo‐inositol (MI) and D‐chiro‐inositol (DCI) administered to PCOS‐model mice at doses comparable to those effective in the management of human patients [18] and in a physiological 40:1 M ratio reflective of human serum levels [19] exerted beneficial effects on ovarian structure and function. In contrast, high doses of DCI, either in combination with MI [16] or alone at supraphysiological levels [17], resulted in abnormal ovarian histology, decreased aromatase expression, altered steroidogenic activity in ovarian cells, and an androgenic phenotype that closely mirrors the clinical features of PCOS patients.

In vitro cultured cells provide a controlled and reproducible alternative for studying steroidogenesis and other PCOS‐related dysfunctions, leading to potential treatments of PCOS to be further preclinically and clinically assessed [15, 20]. While theca cells appear important for investigating features of the disorder that include insulin resistance and luteinising hormone (LH) imbalance, which are involved in the dysregulation of initial steps in steroidogenesis [20], granulosa cells are useful for examining abnormal mechanisms and consequences of androgen‐to‐oestrogen conversion in steroidogenesis. KGN cells are derived from a human granulosa cell tumour and display key physiological characteristics of granulosa cells [21]. Among other features, they respond to FSH stimulation and produce oestrogen, progesterone, and other hormones necessary for follicle development and maturation, thus serving as a valuable model for studying steroidogenesis and molecular alterations associated with PCOS [14, 20, 22].

Based on our previous research on inositols and PCOS in in vivo mouse models, we hypothesized that exposing KGN cells to DCI at concentrations exceeding those typically found in human serum or follicular fluid can induce a PCOS‐like condition and investigate abnormal steroidogenic mechanisms underlying the disorder.

To test our hypothesis, we treated KGN cells with doses of DCI calculated based on the data of Chiu et al. [23], following the observations of Unfer et al. [24], and Sacchi et al. [25], as follows: the concentration of MI in the follicular fluid of healthy women was found to be approximately 30 μM [23], with a molar MI/DCI ratio of 100:1 in healthy individuals and of 0.2:1 in PCOS patients [24]. Based on this, the physiological concentration of DCI in the follicular fluid of healthy women is estimated to be < 30 nM. However, considering the inhibitory effect on aromatase expression observed in human granulosa cells at 20 nM DCI [25], we treated KGN cells with 20 nM, 50 nM, and 100 nM. We used 2 μg/mL of bacterial lipopolysaccharide (LPS) as a positive control for its ability to create a pro‐inflammatory and oxidative environment, and to reduce aromatase expression and activity, resembling PCOS conditions [26, 27, 28]. We used MI at the same concentrations of DCI as a negative control to compare the intrinsic properties of the two isomers. MI was chosen for its beneficial effects on follicle physiology and granulosa cell functions [16, 29, 30].

We assessed cell viability, inflammation, presence of reactive oxygen species, expression of antioxidant genes, expression and activity of the enzyme aromatase. Results confirm general effects of LPS and demonstrate that DCI, but not MI, at the doses employed, significantly reduces aromatase expression and oestrogen secretion, consistent with our previous in vivo findings [17].

## 2 Materials and Methods

### 2.1 Cell Culture

KGN granulosa cells, a kind gift of Prof. Livio Casarini (University of Modena, Modena, Italy) were used in all experiments. Cells were cultured in DMEM‐10% FBS, 4.5 g/L glucose, 1% glutamine, 1% Penicillin–Streptomycin, at 37°C under a humidified atmosphere containing 5% CO_2_. Media were from Euroclone S.p.A. (Pero (MI), Italy).

### 2.2 Cell Treatments

KGN cells were plated in plastic culture flasks (1.5 × 10^6^ cells/75 cm^2^ flask) (Euroclone, Pero (MI), Italy), cultured for 24 h, and then treated by addition of DCI (Amicogen Inc., Jinju‐si, Korea) (final concentrations: 20 nM, 50 nM, 100 nM) LPS (Merck Life Science S.r.l., MI, Italy) (final concentration: 2 μg/mL) or MI (Lo.Li. Pharma, Rome, Italy) (final concentrations: 20 nM, 50 nM, 100 nM) to the culture medium for 24 h, unless otherwise indicated, before further processing. For immunofluorescence analyses and measurements, 5 × 10^3^ cells were plated onto 78.5 mm^2^ coverslips (Epredia, New Hampshire, USA) placed inside 24‐well plates (Euroclone, Pero (MI), Italy) and treated for 24 h as described.

### 2.3 Immunofluorescence Analyses

After treatments, cells were fixed with 4% paraformaldehyde (PFA) (Thermo‐Fisher Scientific, Segrate (MI), Italy) for 10 min at room temperature (RT), washed three times in PBS‐Tween (0.1%) (Tween 20, Merck Life Science S.r.l., MI, Italy), permeabilised in PBS‐Triton (0.1%) (Triton X‐100, Merck Life Science S.r.l., MI, Italy) for 10 min at 4°C, and incubated with a monoclonal mouse anti‐Proliferating Cell Nuclear Antigen (PCNA) primary antibody (1:100) (Thermo‐Fisher Scientific, Segrate (MI), Italy). After an overnight incubation at 4°C, cells were washed three times in PBS‐Tween (0.1%) and incubated with a polyclonal donkey anti‐mouse Dylight 594‐conjugated secondary antibody (1:100) (Bethyl Laboratories, Montgomery, USA) for 1 h at RT. Negative controls were obtained by omitting the primary antibody incubation. To assess total cell numbers, nuclei were stained with 1 μg/mL 4′,6‐diamidino‐2‐phenylindole (DAPI) (Immunological Sciences, Rome, Italy). Blindly, cells in 1 mm^2^ fields were photographed under a Nikon Eclipse 50i microscope (Nikon Instrument S.p.A, Florence, Italy) at 10× magnification, with excitation/emission wavelengths of 594/610 nm and 358/461 nm for PCNA and DAPI, respectively, and counted. Data analysis was performed by calculating the ratio of PCNA+ cells to DAPI+ cells in the treated samples and referring values to those relative to the control samples.

### 2.4 Measurement of Reactive Oxygen Species and Mitochondrial Superoxide

After treatments, CellROX Deep Red Reagent (5 μM) and MitoSOX Red Mitochondrial Superoxide Indicators (1 μM) (Thermo‐Fisher Scientific, Segrate (MI), Italy) were added to the media, and cell staining for reactive oxygen species (ROS) and mitochondrial superoxide (mitoSOX) was performed by incubation for 30 min at 37°C under normal culture conditions, following the manufacturer's instructions. After staining, cells were fixed with 4% PFA for 10 min at RT, and nuclei were counterstained with DAPI (Immunological Sciences, Rome, Italy). Blindly, cells were photographed under a Nikon Eclipse 50i microscope (Nikon Instrument S.p.A, Florence, Italy) at 10× magnification with excitation/emission wavelengths of 644/665 nm for CellROX and 396/610 nm for MitoSOX, and 358/461 nm for DAPI. Approximately 30 cells/treatment were randomly selected from 4 different fields and subjected to fluorescence measurement using ImageJ software (National Institutes of Health, USA).

### 2.5 Gene Expression Analyses

After treatments, 2 × 10^6^ cells were lysed with 1 mL of Trizol Reagent (Thermo Fisher Scientific, Segrate (MI), Italy) and total RNA was isolated according to the manufacturer's protocol. RNA quantification was performed via Multiscan Go (Thermo Fisher Scientific, Segrate (MI), Italy) at a wavelength of 260 nm. RNA purity was evaluated by measuring the ratio 260/280 nm for the proteins. One microgram RNA was retro‐transcribed using an ORIGENE (Herford, Germany) cDNA Synthesis Kit and used as a template for real‐time PCR analyses performed with Taqman Fast Advanced Master Mix (Thermo Fisher Scientific, Segrate (MI), Italy) for qPCR. Analysis of gene expression was performed using TaqMan probes [IL‐6 (Interleukin‐6); GPx1 (Glutathione Peroxidase 1); SOD1 (Superoxide Dismutase 1); SOD2 (Superoxide Dismutase 2); CAT (Catalase); SIRT1 (Sirtuin 1); SIRT3 (Sirtuin 3); CYP19A1 (Aromatase); 18‐S (18S Ribosomal RNA)] (Thermo‐Fisher Scientific, Segrate (MI), Italy), and a common thermal profile of 2 min at 95°C followed by 40 cycles of 95°C for 15 s and 60°C for 30 s. Relative mRNA expression was determined by normalising the amount of cDNA to that of the 18S rRNA and calculated using the 2^−ΔΔCq^ method.

### 2.6 Western Blot Analysis of Aromatase

2 × 10^6^ cells were treated with DCI, MI, and LPS for 24 and 48 h, pelleted and suspended in RIPA lysis buffer 1X containing protease and phosphatase inhibitors (Merck Life Science S.r.l., MI, Italy). Cells were lysed by repeated freezing in liquid nitrogen and thawing, and centrifuged at 16,000 × *g* for 30 min at 4°C. Soluble protein concentration in the supernatant was determined by BCA protein assay kit (Santa Cruz Biotechnology Heidelberg, Germany). Fifteen μg protein from each sample were separated by SDS‐PAGE and transferred to a polyvinylidene difluoride membrane. Non‐specific binding sites were blocked for 1 h at room temperature with 5% milk in Tris‐buffered saline containing 0.1% Tween 20 (TBS‐T). Identification of aromatase and β‐actin was performed by membrane incubation with polyclonal rabbit antibody to aromatase (1:2000) (Antibodies, Cambridge, UK) and β‐actin (1:3000) (Abcam, Cambridge, UK) overnight at 4°C, followed by incubation with horseradish peroxidase (HRP) conjugated anti‐rabbit (1:3000) (Cell Signalling Technology, Danvers, USA) for 1 h at room temperature. After washing in TBS‐T, specific immunoreactive complexes were detected by ECL kit LiteAblot PLUS (Euroclone, Pero (MI), Italy) and Uvitec Cambridge system (Alliance series, Cambridge, UK). Bands were normalised for β‐actin using ImageJ software (National Institutes of Health, USA).

### 2.7 Measurement of Secreted Estradiol Concentrations

Four x10^6^ cells were seeded in 2.5 mL of Phenol Red‐free DMEM‐10% FBS (Euroclone S.p.A., Pero (MI), Italy) in 60 × 15 mm wells. After 24 h, they were treated with 50 nM DCI, 2 μg/mL LPS, or 50 nM MI. After an additional 24 h, 50 nM (or 15 ng/mL) testosterone (T) (Merck Life Science S.r.l., MI, Italy) was added to the cell cultures, and estradiol (E2) secretion was allowed to occur for the following 24 h. Negative control cells were maintained in plain medium for the entire duration of the procedure (72 h), while positive control cells received only the 50 nM testosterone treatment. Cells were finally trypsinized (trypsin from Euroclone, Pero (MI), Italy) and collected by centrifugation at 1000 × *g* for 5 min, counted in a Bürker counting chamber, and lysed for total protein quantification as already described. E2 concentration in 50 μL of cleared culture media was determined by an enzyme‐linked immunosorbent assay kit (CEA461Ge, Cloud‐Clone Corp., USA), according to the manufacturer's instructions.

### 2.8 Statistical Analyses

All experimental procedures were carried out at least three times. Data are presented as means ± standard deviations (SD) of three independent experiments. Statistical analyses were performed with GraphPad Prism 8.0 (GraphPad Software Inc., California, USA) or MedCalc 20.211 (MedCalc Software Ltd., Ostend, Belgium). Based on the assessment of normality and homogeneity of variance using the Shapiro–Wilk test, the effects of LPS were compared using Student's t‐test, while the effects of various doses of DCI and MI were analysed by one‐way ANOVA. *p* < 0.05 was considered statistically significant.

## 3 Results

### 3.1 DCI, LPS and MI do Not Affect Proliferation and Survival of KGN Cells

**FIGURE 1:**
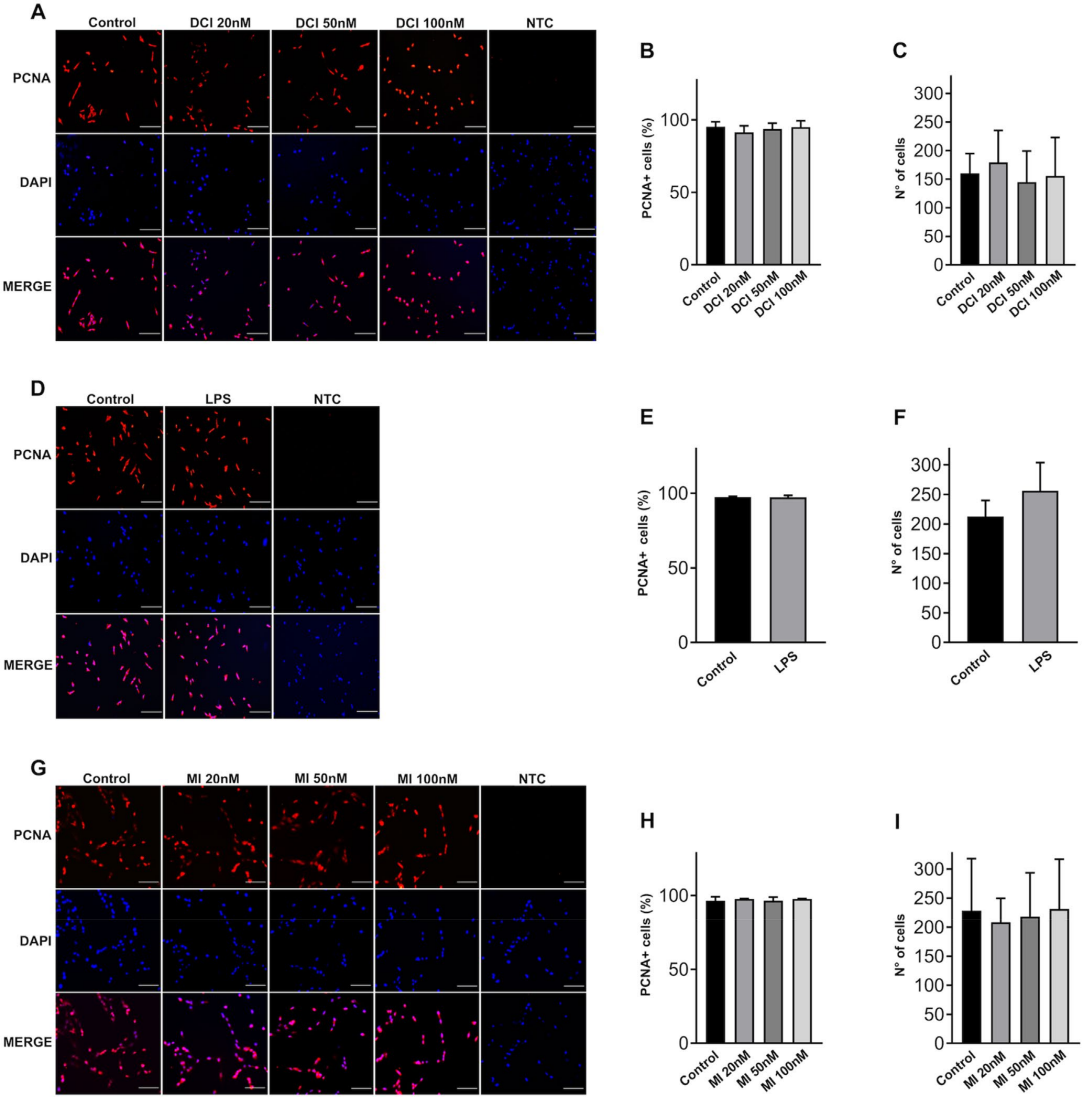
Effects of DCI, LPS and MI on KGN cell survival and proliferation. (A, D, G) Representative immunofluorescence and DAPI staining images after DCI, LPS or MI treatments for 24 h; NTC, negative control, scale bars = 100 μm; (B, E, H) analysis of KGN cell proliferation after treatments with DCI, LPS or MI for 24 h using anti‐PCNA antibody; (C, F, I) number of viable cells after treatments with DCI, LPS or MI for 24 h. Data are the mean ± SD of 3 independent experiments. LPS vs. control, Student's test; DCI and MI vs. control, one‐way ANOVA.

The effects of DCI (Figure 1A,B), LPS (Figure 1D,E) and MI (Figure 1G,H) on KGN cell proliferation were evaluated by immunofluorescence analysis of the abundance of PCNA, a marker highly expressed in proliferating cells and involved in DNA replication and cell cycle progression. The results showed no differences between PCNA‐positive cells among all samples. These findings suggest that DCI, MI and LPS do not affect KGN cell proliferation after 24 h of in vitro treatment. The potential cytotoxic effects of DCI, LPS and MI were evaluated by labeling nuclei with DAPI following cell fixation in 4% PFA. Cell viability was assessed by comparing the number of nuclei in cells treated for 24 h with DCI, LPS and MI (Figure 1C,F,I) with those of control cells. Various treatments did not affect KGN cell survival, suggesting absence of cytotoxicity in vitro.

### 3.2 DCI Has Antioxidant Effects in KGN Cells

**FIGURE 2:**
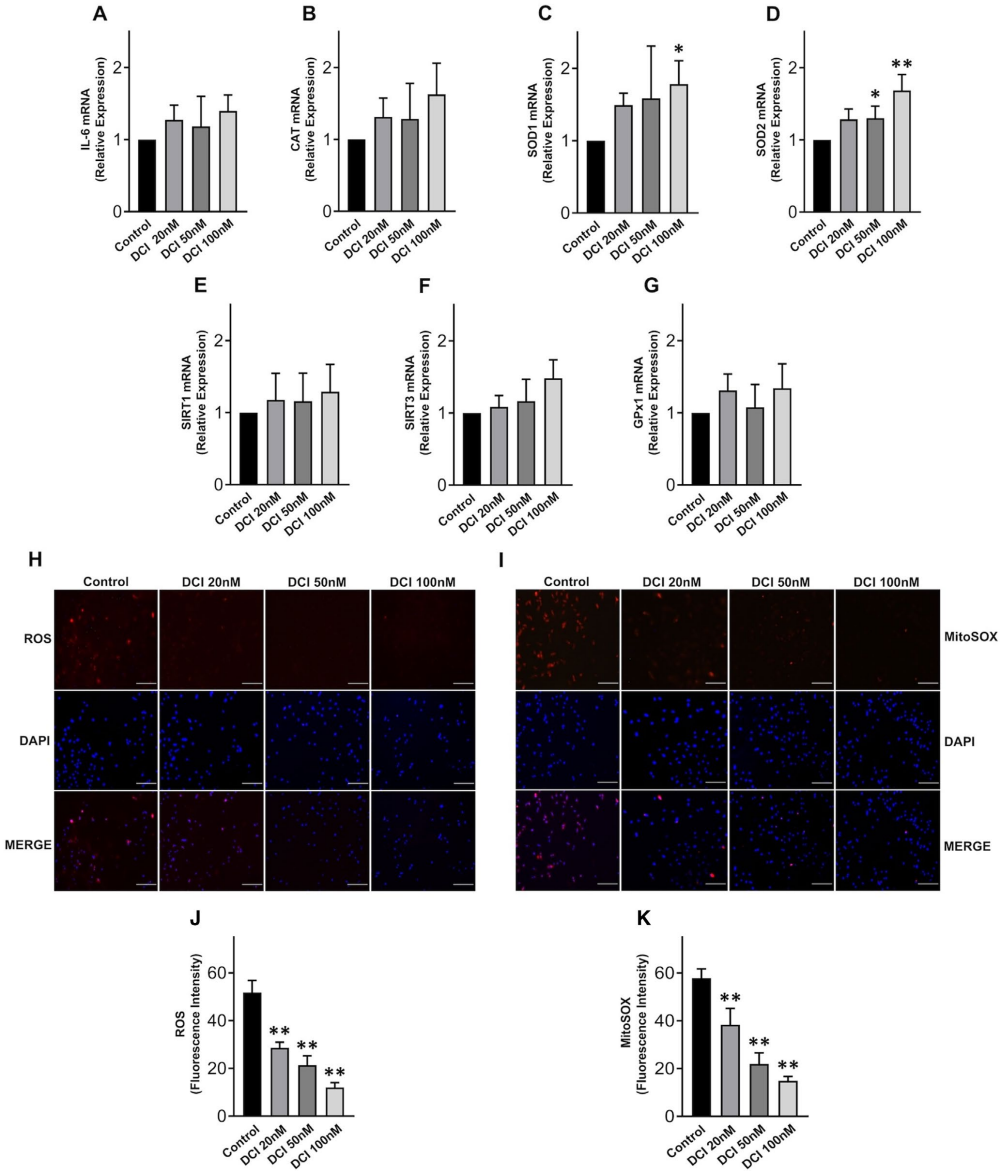
Antioxidant effects of DCI in KGN cells. Levels of mRNA expression of (A) IL‐6, (B) CAT, (C) SOD1, (D) SOD2, (E) SIRT1, (F) SIRT3, (G) GPx1 after DCI treatments for 24 h; (H, I) representative images of fluorescence and DAPI staining after DCI treatments for 24 h; scale bar = 100 μm; (J) ROS and (K) mitochondrial superoxide (mitoSOX) measurement after DCI treatments for 24 h. Data are the mean ± SD of 3 independent experiments. *p < 0.05; **p < 0.01, one‐way ANOVA.

Treatments of KGN cells with DCI did not modify IL‐6 expression (Figure 2A) and suggested a trend toward an increase in mRNA expression of antioxidant genes (Figure 2B–G) with a notable upregulation of SOD1 at 100 nM (Figure 2C) and of SOD2 at 50 nM and 100 nM (Figure 2D). These findings are consistent with the observed significant reductions in ROS (Figure 2H,J) and mitochondrial superoxide (Figure 2I,K) levels, suggesting that DCI exerts antioxidant effects. With respect to control cells, ROS levels were reduced by 44.6% by 20 nM DCI, 58.9% by 50 nM DCI, and 76.7% by 100 nM DCI (*p* < 0.0001 in all cases, one way ANOVA); mitochondrial superoxide levels were reduced by 34.3% by 20 nM DCI, 62.8% by 50 nM DCI, and 74.4% by 100 nM DCI (*p* < 0.0001, in all cases, one way ANOVA).

### 3.3 LPS Induces Inflammatory and Oxidative Stress Responses in KGN Cells

**FIGURE 3:**
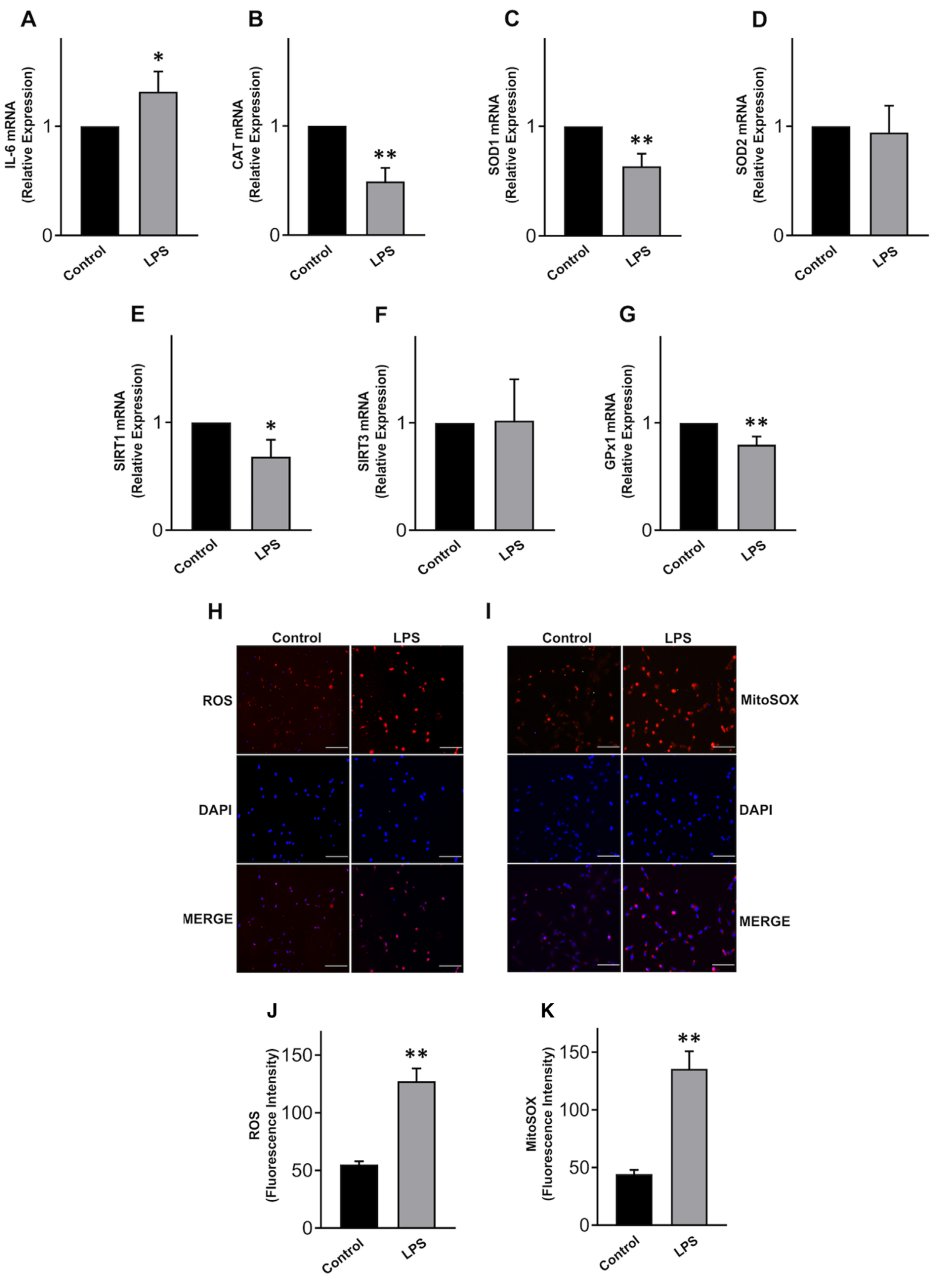
Inflammation and oxidative stress induced by LPS in KGN cells. Levels of mRNA expression of (A) IL‐6, (B) CAT, (C) SOD1, (D) SOD2, (E) SIRT1, (F) SIRT3, (G) GPx1 after LPS treatments for 24 h; (H, I) representative images of fluorescence and DAPI staining after LPS treatments for 24 h; scale bar = 100 μm; (J) ROS and (K) mitochondrial superoxide (mitoSOX) measurement after LPS treatments for 24 h. Data are the mean ± SD of 3 independent experiments. *p < 0.05; **p < 0.01, Student's test.

Treatment of KGN with LPS induced an inflammatory response, as evidenced by the significant increase in IL‐6 mRNA expression (Figure 3A). Additionally, there was a significant downregulation of antioxidant genes such as CAT, SOD1, GPx1, and SIRT1 (Figure 3B–G), while no significant changes were observed for SOD2 and SIRT3 genes (Figure 3D,F). Since the downregulation of antioxidant genes is associated with oxidative stress, these results align with the observed increase in both ROS (Figure 3H,J) and mitochondrial superoxide (Figure 3I,K) levels of 131.0% and 204.8%, respectively (ROS *p* < 0.0001; mitochondrial superoxide *p* < 0.0001, Student's‐test). Taken together, these findings confirm the previously described inflammatory and oxidative effect of LPS [27].

### 3.4 MI Has Antioxidant Effects in KGN Cells

**FIGURE 4:**
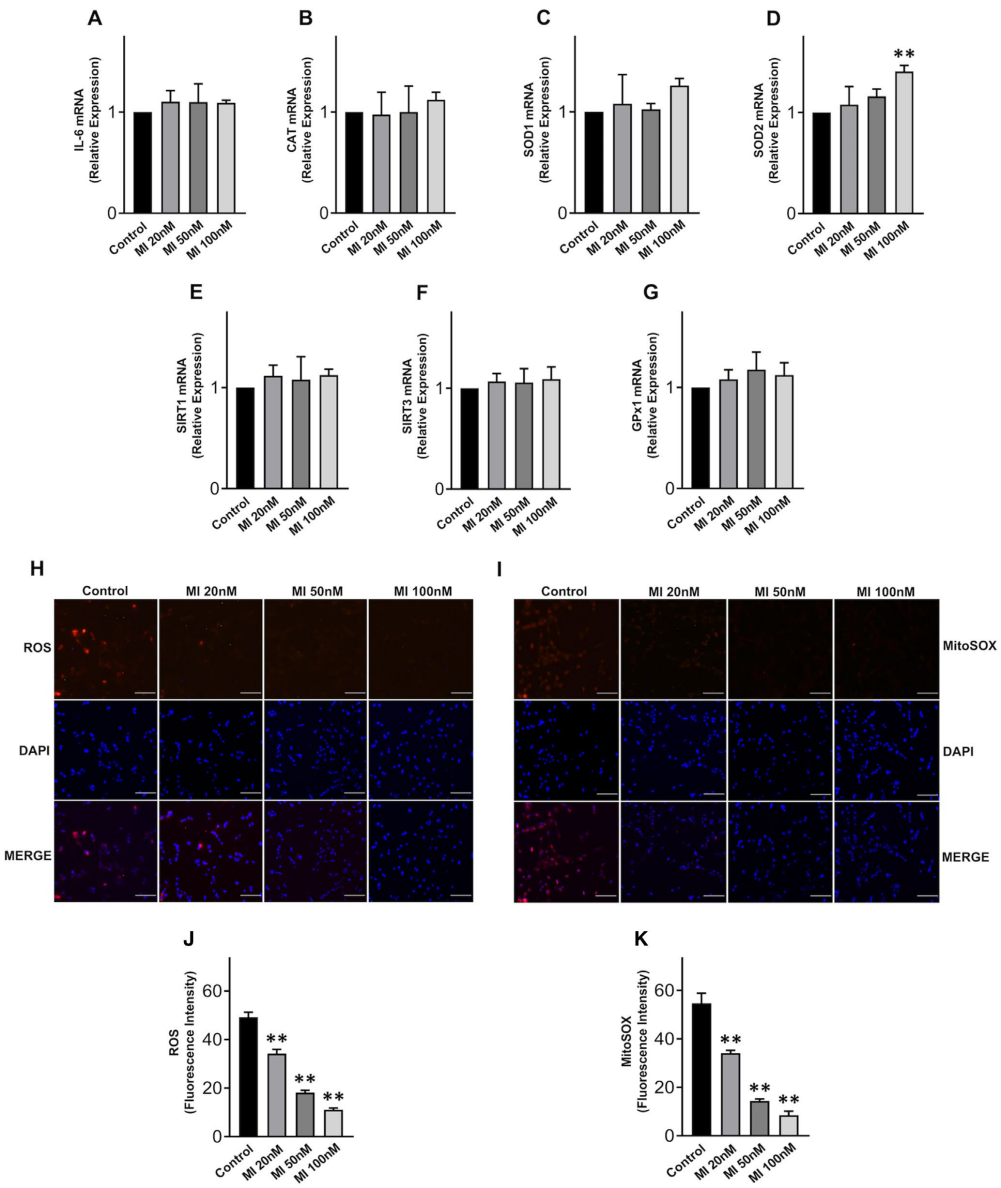
Antioxidant effects of MI in KGN cells. Levels of mRNA expression of (A) IL‐6, (B) CAT, (C) SOD1, (D) SOD2, (E) SIRT1, (F) SIRT3, (G) GPx1 after MI treatments for 24 h; (H, I) representative images of fluorescence and DAPI staining after MI treatments for 24 h; scale bar = 100 μm; (J) ROS and (K) mitochondrial superoxide (mitoSOX) after MI treatments for 24 h. Data are the mean ± SD of 3 independent experiments. **p < 0.01, one‐way ANOVA.

Like DCI, treatments of KGN with MI did not modify the expression of IL‐6 (Figure 4A), had an antioxidant effect, increasing the expression of SOD2 (Figure 4D), but not other antioxidant genes (Figure 4B,C,E–G), and strongly reducing ROS (Figure 4H,J) and mitochondrial superoxide (Figure 4I,K) levels. With respect to control cells, ROS levels were reduced by 30.5% by 20 nM MI, 62.9% by 50 nM MI and 77.5% by 100 nM MI (*p* < 0.0001 in all cases, one way ANOVA); mitochondrial superoxide levels were reduced by 37.4% by 20 nM MI, 73.5% by 50 nM MI and 84.4% by 100 nM MI (*p* < 0.0001, in all cases, one way ANOVA). These results are consistent with an anti‐inflammatory action of MI [31] and confirm its previously described antioxidant effects [29].

### 3.5 DCI and LPS, but Not MI, Reduce *CYP19A1* Expression in KGN Cells

**FIGURE 5:**
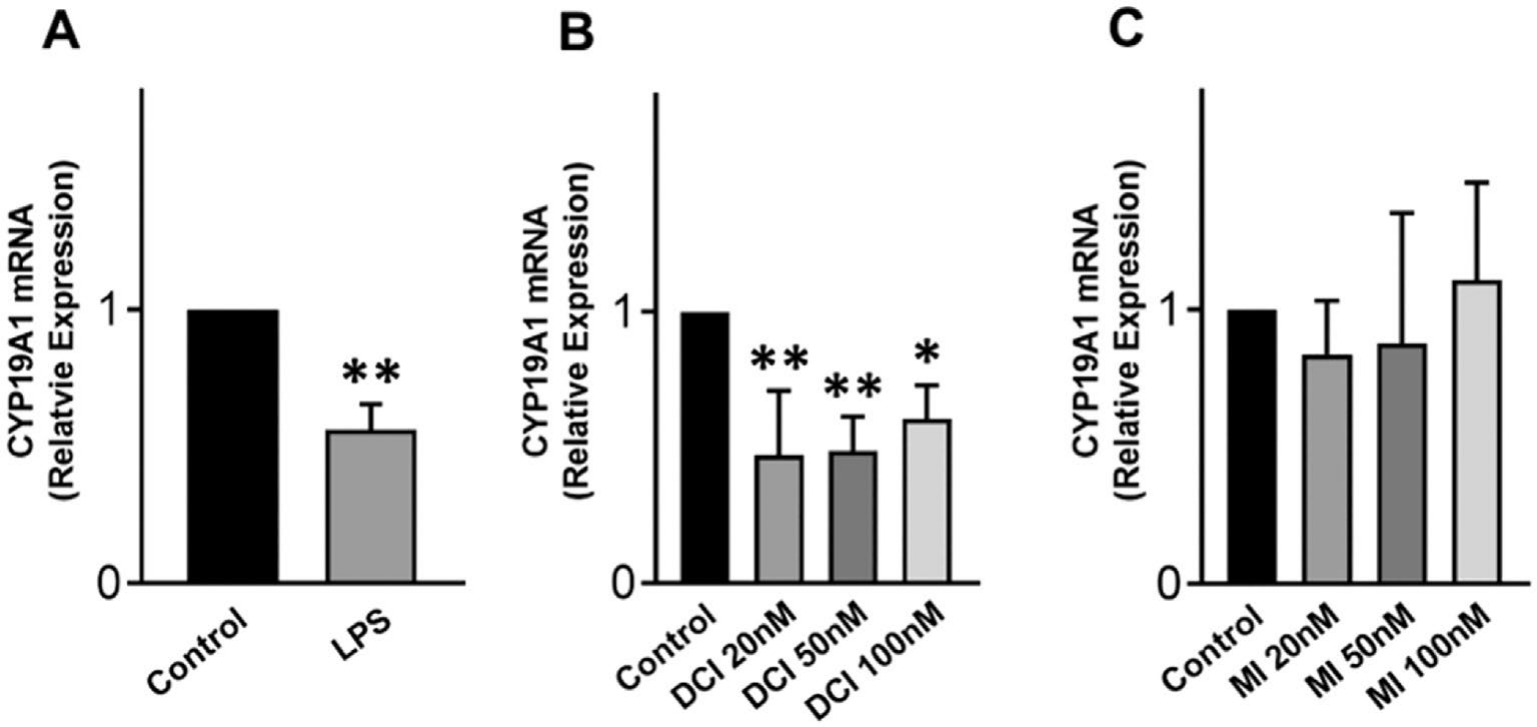
*CYP19A1* mRNA expression in KGN cells after DCI, LPS or MI treatment. Levels of aromatase mRNAs after (A) LPS, (B) DCI or (C) MI treatments for 24 h. Data represent the mean ± SD of 3 independent experiments; *p < 0.05; **p < 0.01. LPS vs. control, Student's test; DCI and MI vs. control, one‐way ANOVA.

Treating KGN cells for 24 h with LPS and DCI, but not MI, significantly reduced the amount of *CYP19A1* gene transcripts (Figure 5A–C). These findings confirm the observations of Sacchi et al. [25] and highlight the following points: (a) despite its anti‐inflammatory and antioxidant properties, DCI downregulates aromatase expression; (b) in addition to its pro‐inflammatory and oxidative effects, LPS also reduces aromatase expression, confirming previous reports [26, 27]; and (c) MI confirms its well‐known beneficial effects on granulosa cell functions by not altering aromatase expression.

### 3.6 DCI and LPS, but Not MI, Reduce Levels of Aromatase Protein in KGN Cells

**FIGURE 6:**
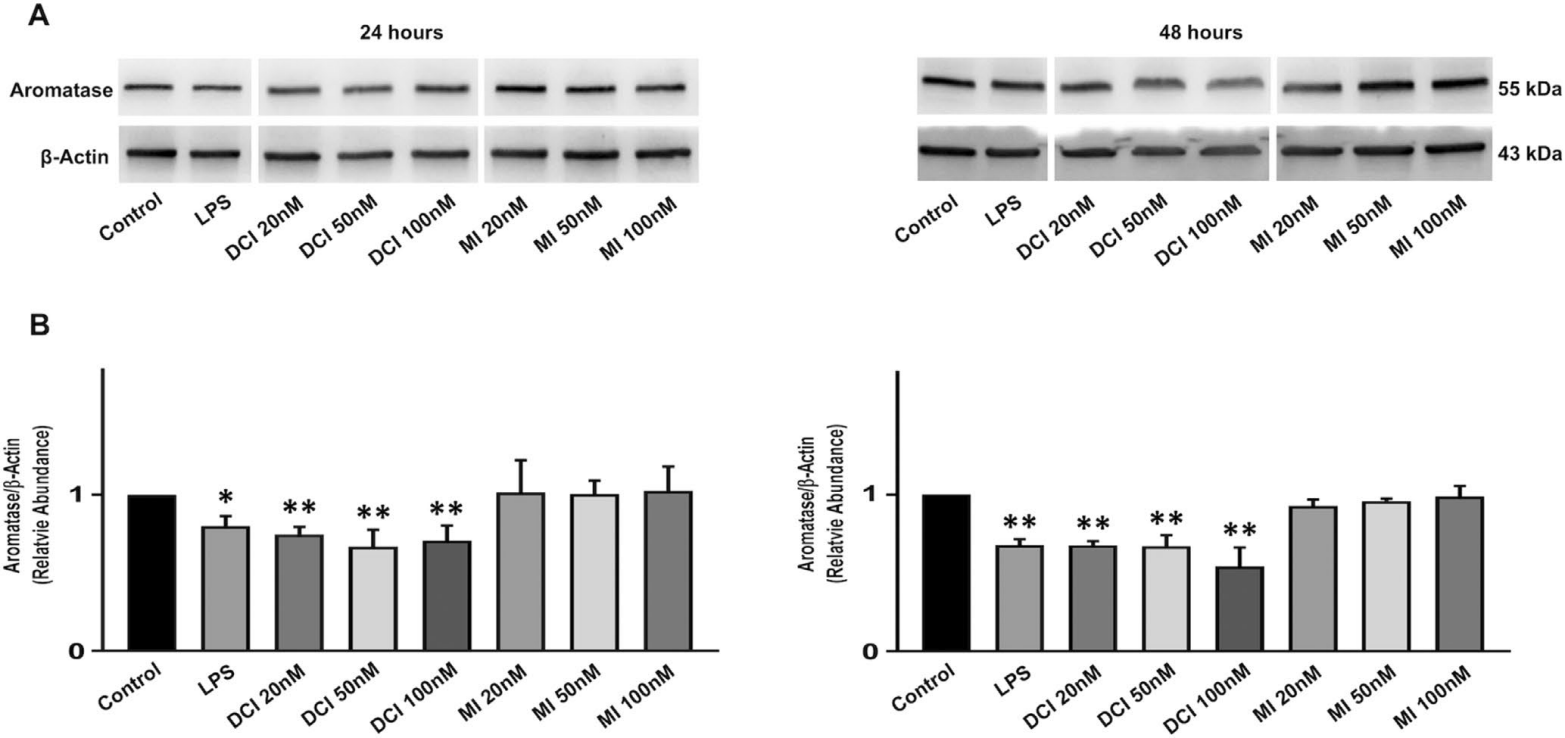
Aromatase protein levels in KGN cells after DCI, LPS or MI treatment. (A) Representative Western blot of aromatase and β‐Actin in protein extracts from KGN cells after DCI, LPS or MI treatments for 24 h (left) or 48 h (right); (B) densitometric analysis of aromatase/β‐Actin levels after DCI, LPS or MI treatments for 24 h (left) or 48 h (right). Data represent the mean ± SD of 3 independent experiments. **p < 0.01. LPS vs. control, Student's test; DCI and MI vs. control, one‐way ANOVA.

The abundance of aromatase relative to β‐actin in KGN cells was evaluated after treatments with LPS, DCI, and MI for 24 and 48 h (Figure 6A) by Western blot analysis followed by densitometric quantification. Electrophoretic separation of extract proteins revealed a distinct 55 kDa band for aromatase, as already reported [32]. Densitometric analysis of aromatase band intensities revealed a decrease in cells treated with LPS and DCI at 24 and 48 h (Figure 6B), while no significant changes were observed following MI treatment. These findings confirm mRNA expression data, providing further evidence that DCI reduces protein levels of aromatase in KGN cells, in line with what was observed in mouse ovaries in vivo [17]. They also confirm previous observations on LPS [26] and show that MI does not affect aromatase expression both at the mRNA and at the protein level.

### 3.7 DCI and LPS, but Not MI, Reduce Estradiol Secretion in KGN Cells

**FIGURE 7:**
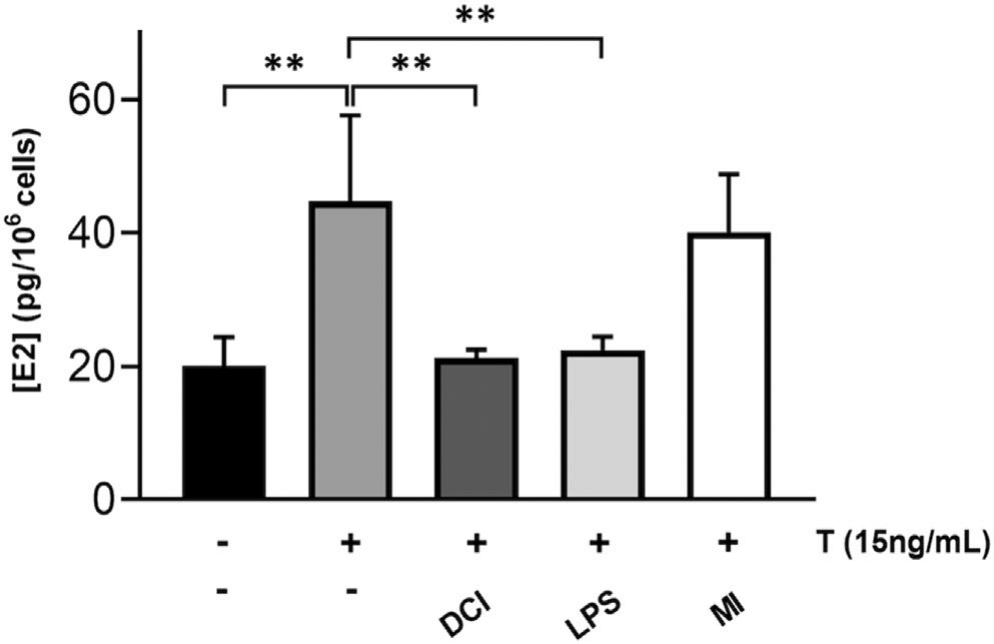
Effects of DCI, LPS and MI on KGN cell estradiol secretion. Concentration levels of E2 secreted by KGN cells cultured in DCI, LPS or MI for 48 h and provided with 50 nM testosterone for the final 24 h. Data represent the mean ± SD of 3 independent experiments. **p < 0.01; one‐way ANOVA.

The effects of 50 nM DCI, 2 μg/mL LPS, and 50 nM MI on testosterone‐to‐estradiol conversion in KGN cells were finally evaluated. As for DCI and MI, the intermediate 50 nM dose was used. Experimental conditions included measurements of basal estradiol synthesis in the absence of testosterone supplementation (control −). In all other conditions, cells were supplemented with 50 nM (or 15 ng/mL) testosterone. This induced intense estradiol secretion in KGN cells (control +), at levels significantly above those observed in control‐cells (Figure 7). In contrast, supplementation of testosterone to cells maintained in the presence of 50 nM DCI or 2 μg/mL LPS resulted in a significant reduction in estradiol levels (Figure 7). This finding confirms that both compounds reduce overall aromatase activity, thereby disrupting the androgen‐oestrogen balance and inducing an androgenic phenotype. Conversely, stimulation with MI did not cause significant changes in estradiol levels, supporting the hypothesis that 50 nM MI does not affect aromatase expression and estradiol secretion (Figure 7).

## 4 Discussion

PCOS is a serious endocrine disorder characterised by hormonal imbalances, oxidative stress, chronic inflammation, infertility, and metabolic disturbances, with a significant impact on the quality of life of affected patients [3]. Considering the absence of optimal therapeutic options, the need for preclinical research and the development of reliable models to study the underlying pathogenic mechanisms and key pathways of the syndrome becomes crucial.

In our previous work, we investigated ovarian function and its alterations in PCOS using in vivo mouse models [17]. In the present study, we treated KGN cells with DCI or LPS and analysed their effects on inflammation, levels of reactive oxygen species, aromatase expression and activity in an attempt to establish in vitro models of PCOS. In our assessments, we used MI as the negative control for both DCI and LPS effects.

DCI did not induce overexpression of IL‐6, suggesting no pro‐inflammatory effect, and exhibited antioxidant properties by increasing the expression of antioxidant genes such as SOD1 and SOD2 along with a parallel reduction of ROS and mitochondrial superoxide levels [33]. This aligns with the described free radical scavenging properties of DCI, which contribute to the reduction of oxidative stress and the improvement of cellular function [33, 34]. Zhang et al. [35] demonstrated that DCI can activate the Nuclear Factor Erythroid 2‐Related Factor 2 (NRF2), a transcription factor that binds to antioxidant response elements and promotes transcription of antioxidant genes [36]. The ability to regulate NRF2 may explain the induction of genes coding for SOD1 and SOD2, which contribute to reducing the levels of oxygen radicals in the cells.

We can rule out the possibility that DCI induces a reductive stress in cells not undergoing oxidative stress, as the treatments had no effects on cell proliferation and viability. However, further investigation into mechanisms underlying the cellular action of different doses of DCI and the NADH/NAD^+^ balance [37] under various conditions is needed to clarify this issue.

Despite these apparently beneficial cellular effects, DCI produced a specific reduction in aromatase expression, resulting in a decrease in estradiol secretion. This suggests a negative impact on the androgen–oestrogen balance, further confirming observations of Sacchi et al. [25] and our group [17], which were respectively obtained in human in vitro cultured cumulus cells and our mouse PCOS‐like model. The inhibitory effect of DCI on aromatase expression is likely mediated by the modulation of the Phosphoinositide 3‐Kinase/protein Kinase‐B (PI3K/AKT) pathway, which regulates expression of the *CYP19A1* gene in various cell types [13, 38]. DCI is known to increase the expression of Insulin Receptor Substrate 2, PI3K and AKT, upregulating the level of phosphorylated AKT, and downregulating the levels of the Glycogen Synthase Kinase 3 beta protein [39]. While these mechanisms are crucial for regulating insulin signalling and are essential for the function of glycogen‐synthesising cells, i.e., liver or muscle cells, they also appear to play a central role in the inhibition of aromatase expression in granulosa cells.

LPS, used as a positive control [27], elicited a typical cellular response to bacterial infection, triggering inflammatory and oxidative reactions including an increase in IL‐6 expression, a reduced expression of antioxidant genes, and an increase in ROS and mitochondrial superoxide levels. In addition, LPS downregulated aromatase expression and consequently reduced enzyme activity. This effect is consistent with the described ability of LPS to activate the Toll‐like receptor 4, which modulates the expression of genes involved in steroidogenesis in granulosa cells [26, 28]. In the absence of defined effects on cell proliferation, LPS's pleiotropic and toxic action on KGN cells provides a model for studying the inflammatory and oxidative alterations associated with PCOS [26, 27] and other ovarian conditions such as ovarian aging [40]. To this respect, LPS has been shown to negatively affect folliculogenesis in vivo via the PI3K/Phosphatase and Tensin Homologue/AKT/Forkhead Box O3 signalling pathway [41].

MI, the major DCI isomer, was used as the negative control in our experiments. MI is present at micromolar concentrations in the follicular fluid of fertile women [23] and has been shown to have beneficial effects on the metabolic profile of mouse oocytes [29] and reproductive functions when administered to women with PCOS [42]. As expected, MI treatment had no effects on IL‐6 expression and exhibited antioxidant properties [43], increasing the expression of SOD2 and reducing ROS and mitochondrial superoxide levels. In contrast to DCI, MI did not affect aromatase activity, confirming its physiological role in granulosa cells and ovarian function [44].

These results complement those obtained with DCI and LPS and underscore the therapeutic potential of MI in managing PCOS. MI has been shown to support follicular development, enhance the viability of oocyte/cumulus cell complexes [29], and promote oestrogen biosynthesis without disrupting the androgen–oestrogen balance [30].

The results of present experiments appear to contrast with the findings of Wojciechowska et al. [45], who reported that aromatase mRNA expression in KGN cells is reduced by 1 mM MI and unaffected by 20 nM DCI. However, several critical points should be considered concerning that study:

- the concentration of MI used is 10–50 times higher than the doses employed in our experiments;
- their conclusions are based solely on analyses of *CYP19A1* transcripts, without corroborating evidence from Western blot evaluations of aromatase protein levels;
- the control transcript levels in their RT‐qPCR graphs are not normalised to 1.0 ([45], Figure 6);
- no reduction in estradiol secretion was observed with 1 mM MI, which would be expected if aromatase expression was indeed reduced ([45], Figure 7);
- a reduction in estradiol secretion was observed with 20 nM DCI, even in the absence of decreased aromatase expression ([45], Figure 7);
- a more pronounced reduction in estradiol secretion was observed with the combination of 1 mM MI and 20 nM DCI, despite no corresponding decrease in aromatase expression ([45], Figure 7).

Altogether, methodological and technical aspects of that publication cast serious doubts regarding: (a) the correct interpretation of the results; and (b) their opposite direction toward the current knowledge on the effects of both MI and DCI on follicle dynamics and the use of these molecules in the treatment of PCOS patients.

## 5 Working Hypotheses and Future Perspectives

Regarding the inhibitory effect on *CYP19A1*, it is possible that high levels of DCI disrupt the PI3K/AKT pathway, leading to transcriptional repression of specific genes through regulatory mechanisms that may involve epigenetic modifications, such as promoter CpG island methylation. Given that a CpG island is present in the promoter region of CYP19A1 [46], we hypothesise that this region may be a target of DNA methyltransferases, mediating the inhibitory effect of DCI on aromatase expression. The effects of DCI on the modulation of the PI3K/AKT pathway, along with other cellular pathways, and the precise nature of its inhibitory action on CYP19A1 expression are currently under investigation in our laboratory.

The partial convergence of the signal transduction pathways of DCI and LPS on one hand, and of MI and DCI on the other, may represent a key avenue for future research on the aetiology of PCOS.

In this context, the convergence of cellular pathways regulated by MI and DCI, including serine phosphorylation of AKT [47, 48], threonine/tyrosine phosphorylation of mitogen‐activated protein kinase/extracellular signal‐regulated kinase 1‐2 [48], and the balance between these signalling events under both physiological and pathological conditions, appears particularly intriguing.

Our results may help in interpreting our previous observations in the mouse PCOS‐like model. In that study, cystic follicles were observed following treatments with both DCI at high doses and the aromatase enzyme inhibitor letrozole [17]. Based on our novel findings, we hypothesise that, despite inflammation, oxidative stress and other metabolic disturbances associated with PCOS, ovarian cysts may be the direct and exclusive consequence of the hyperandrogenic phenotype, which is linked to the downregulation of CYP19A1 expression in granulosa cells. Given that a dramatic increase in DCI concentration in the follicular fluid from PCOS patients is well described [24], we extend our hypothesis, verifiable in ovarian samples from IVF cycles, to suggest that a DCI‐induced reduction in aromatase activity in granulosa cells below a certain threshold may represent the initial step in the development of degenerative ovarian follicles. This process could occur independently of other PCOS hallmarks, such as oxidative stress and inflammation.

## 6 Conclusions and Limitations

In conclusion, we demonstrate that PCOS‐like models can be established in human KGN granulosa cells to study cellular alterations likely central to the syndrome. Specifically, our results show that DCI and LPS, at the doses used in our experimental conditions, affect cellular functions central to PCOS. While LPS induces alterations at multiple levels, the effect of DCI appears limited to the induction of a hyperandrogenic PCOS‐like phenotype through the inhibition of aromatase expression and activity.

Since PCOS is characterised by hyperandrogenism, inflammation and oxidative stress, each of which is induced by LPS, our findings suggest treating KGN cells with this complex compound can provide deeper insight into cellular pathways affecting granulosa cell metabolism and overall function. Altering these pathways may eventually lead to a reduction in steroidogenic activity, oestrogen production, and to a consequent failure in folliculogenesis. This model could be crucial for identifying specific biomarkers associated with oxidative stress and inflammation, as well as for assessing their impact on steroidogenesis.

In contrast, treatment of KGN cells with DCI produces a model specifically focused on the reduction of aromatase activity. This finding is particularly significant as it targets the endpoint of cellular alterations that lead to impaired steroidogenic activity and hyperandrogenism, allowing for the study of abnormal transcriptional regulatory mechanisms central to PCOS.

Looking forward, our in vitro model(s) could improve the understanding of the cellular pathophysiology of PCOS and support the development of therapeutic treatments for women affected by the syndrome.

Present results further confirm the clinical implications of the mouse PCOS‐like model [17]. The observed effect of DCI must always be considered when administering this molecule to hyperandrogenic PCOS patients at high doses or over extended periods. In fact, while DCI may offer metabolic benefits, it would also exacerbate hyperandrogenism and worsen PCOS symptoms.

This study carries the inherent limitations associated with the use of tumour‐derived granulosa cells, which may exhibit genetic mutations and/or transcriptional alterations that can lead to atypical signalling pathways or responses to drugs and stress conditions. However, increasing evidence, including the present findings, suggests that these cells represent a valuable tool for investigating specific metabolic processes and for developing in vitro models of human ovarian diseases. In the context of translational research, extending these conclusions to more physiologically relevant preclinical models will require direct comparisons with both primary granulosa cell cultures and in vivo mouse models. These objectives are currently being pursued in our laboratory.

## Author Contributions

**Cristiano Giuliani:** conceptualization (equal), data curation (lead), investigation (lead), methodology (equal), writing – original draft (lead), writing – review and editing (supporting). **Giovanni Casoli:** investigation (supporting). **Giovanna Di Emidio:** methodology (supporting), writing – review and editing (supporting). **Carla Tatone:** conceptualization (supporting), funding acquisition (supporting), methodology (supporting), writing ‐ review and editing (supporting). **Arturo Bevilacqua:** conceptualization (lead), data curation (equal), funding acquisition (lead), methodology (equal), project administration (equal), supervision (equal), validation (equal), writing – original draft (equal), writing – review and editing (lead).

## Ethics Statement

The authors have nothing to report.

## Consent

The authors have nothing to report.

## Conflicts of Interest

The authors declare no conflicts of interest.

## Data Availability

The datasets used or analysed during the current study are available from C.G. or A.B. upon reasonable request.
